# Metabolic and Demographic Feedbacks Shape the Emergent Spatial Structure and Function of Microbial Communities

**DOI:** 10.1371/journal.pcbi.1003398

**Published:** 2013-12-26

**Authors:** Sylvie Estrela, Sam P. Brown

**Affiliations:** 1 Institute of Evolutionary Biology, School of Biological Sciences, University of Edinburgh, Edinburgh, United Kingdom; 2 Centre for Immunity, Infection & Evolution, University of Edinburgh, Edinburgh, United Kingdom; University of Chicago, United States of America

## Abstract

Microbes are predominantly found in surface-attached and spatially structured polymicrobial communities. Within these communities, microbial cells excrete a wide range of metabolites, setting the stage for interspecific metabolic interactions. The links, however, between metabolic and ecological interactions (functional relationships), and species spatial organization (structural relationships) are still poorly understood. Here, we use an individual-based modelling framework to simulate the growth of a two-species surface-attached community where food (resource) is traded for detoxification (service) and investigate how metabolic constraints of individual species shape the emergent structural and functional relationships of the community. We show that strong metabolic interdependence drives the emergence of mutualism, robust interspecific mixing, and increased community productivity. Specifically, we observed a striking and highly stable emergent lineage branching pattern, generating a persistent lineage mixing that was absent when the metabolic exchange was removed. These emergent community properties are driven by demographic feedbacks, such that aid from neighbouring cells directly enhances focal cell growth, which in turn feeds back to neighbour fecundity. In contrast, weak metabolic interdependence drives conflict (exploitation or competition), and in turn greater interspecific segregation. Together, these results support the idea that species structural and functional relationships represent the net balance of metabolic interdependencies.

## Author Summary

Understanding the structure and functioning of polymicrobial communities is a major challenge in biology, as witnessed by the dramatic yet mysterious roles played by the human microbiome in human health. Microbial multispecies communities often show complex spatial structures and patterns of metabolic exchange, yet our understanding of how species spatial and ecological relationships emerge from the metabolic rules of species interactions is still limited. What mechanisms underlie multispecies community self-organization? In this study, we simulate the growth of a minimal—two species— community and show how the emergent properties of community spatial structure and function depend on the nature of metabolic interactions between the two species. We found that strong mutual need for help (strong metabolic interdependence) favours the emergence of mutualism, increased productivity and lineage mixing via striking and highly stable branching patterns. In contrast, when the mutual need for help is low, conflict dominates and the two species tend to segregate. Finally, we show how the emergent species mixing follows from a positive feedback of providing aid to neighbouring helpers.

## Introduction

It is now widely accepted that most polymicrobial communities living in natural environments form spatially structured and surface-attached consortia (biofilms) [1]. There has recently been a great interest in investigating how spatial structure may forge and stabilize the complex web of interactions occurring within these multispecies communities, including mutualistic [2] and competitive [3] relationships. Empirical work in multispecies biofilms has acknowledged that species composition affects community structure and species distribution within the biofilm [4] as a result, for example, of mixing species that have distinct monoculture structures [5], or via metabolic interactions, such as cross-feeding [6], [7], [8], [9] or detoxification of exogenous waste products [10]. The type of carbon source also plays a major role in generating the diversity of spatial arrangements observed in polymicrobial communities, as varying the source of carbon likely alters the metabolic interactions between members of the community. For example, in a two-species biofilm consisting of *Burkholderia* and *Pseudomonas*, Nielsen et al. [8] observed that when the two species were competing for a common resource (non-cross-feeding medium), the biofilm consisted of separate microcolonies (high species segregation). In contrast, when the two species were involved in a one-way obligate cross-feeding interaction (cross-feeding medium), the microcolonies consisted of both species (greater mixing).

Evolutionary theory has suggested that spatial mixing favours the evolution of mutualism because it keeps mutualistic partners in close proximity, thereby allowing for stronger reciprocity [11], [12], [13], which may in turn facilitate the exchange of metabolites between partners. However, it has also been proposed that, under some conditions, spatial mixing may impair mutualism because of spatial limits on exchange [14], or because it hinders cooperators' clustering in within-species cooperation [15]. Empirical work on the evolution of microbial cross-feeding mutualisms has also found opposite responses to environment structure. For example, Harcombe [16] provided empirical support for the benefits of spatial structure in the evolution of mutualistic cross-feeding between *Salmonella* and auxotrophic *Escherichia coli*. However, another study on the nascent cross-feeding mutualism between *Desulfovibrio vulgaris* and *Methanococcus maripaludis* showed that mutualism was initially favoured in a well-mixed rather than static environment [17]. Although the authors suggested that this different response to environmental structure is due to the lack of a direct fitness cost of cooperation in the latter cross-feeding model system [18], other mechanisms may be at play as well. The spatial separation (distance) between species has also been identified as a key factor for the stable coexistence of a synthetic mutualistic bacterial community [19].

While evolutionary ecology has traditionally assumed that structure is a fixed environmental property (i.e. either structured or well-mixed), there has been a recent interest in regarding structure as an emergent property of the aggregate behaviour of individuals [20]). Individual-based simulations of microbial growth have started to shed some light on this topic. For example, Nadell et al. [21] explored how physical and biological parameters of bacterial growth in biofilm affect lineage segregation, which in turn determines the fate of within-species cooperation. Using the same framework, it has also been proposed that within-species cooperation can be favoured due to social insulation of cooperators from non-cooperators by a second species [22]. Recently, using a mix of experiments and simulations, Momeni et al. [23] showed that strong inter-population cooperation led to inter-population mixing in microbial communities, and specifically in a pattern of successive layering. Despite this, however, far too little attention has been given to how specific metabolic interactions generate the emergent spatial and functional properties of microbial communities.

Our goal here is to address this question by investigating how metabolic constraints of individual species shape the emergent functional relationships and spatial structuring of a two-species community. For this, we focus on a specific type of interspecific metabolic interaction - trading food for detoxification (for empirical examples see [24], [17]; for a theoretical approach see [25]), and we explore how a partner's need for help (either detoxification to the producer or food to the cross-feeder) affects the ecology, spatial structure, and productivity of the two-species community.

Using an individual-based modeling (IBM) framework that models microbial population growth on a solid surface [26], our results show that stronger metabolic interdependence generates more mutualism, more interspecific mixing, less sensitivity to initial conditions and enhanced community productivity. The emergence of this metabolism-dependent community structure and functioning is driven by demographic feedbacks, such that providing aid to a mutualistic partner generates a positive feedback on the individual's growth whereas providing aid to a competitor or exploiter generates a negative feedback on the individual's growth. In consequence, demographic feedbacks strengthen mutualistic relationships via increased lineage mixing, and weaken competitive relationships via increased segregation.

## Results

We model the growth of a two-species microbial community on an inert surface using an individual-based modeling (IBM) framework described in detail in Lardon et al. [26]. Individual-based models have proven useful in addressing ecological and evolutionary questions in biofilms and are a powerful approach to study the emergent properties of microbial communities [21], [22], [27], [28], [29], [30], [31]. Briefly, this framework simulates the growth of bacterial cells on a surface that grow by consuming nutrients present in their local environment, and then divide. The transport of solutes into and within the biofilm occurs through diffusion, which is assumed to occur much faster than cell growth and division. Cell movement within the biofilm occurs as a result of cell growth, division, shrinking and death. Bacterial cells interact mechanically with neighbouring cells by shoving for space, a process that minimizes cells overlap. Metabolic interactions are introduced by the explicit modeling of metabolic intermediates, subject to defined stoichiometry of metabolic reactions and rates of diffusion (Table S1 and Methods, and for more details on the assumptions of the IBM framework see [26]).

### Metabolic interdependence shapes emergent functional relationships

The ecological outcome of the food for detoxification interaction depends on the balance between costs and benefits of interspecific association. The potential costs are interspecific competition for common nutrients and space, while the potential benefits are food for the cross-feeder and detoxification for the producer. To determine the type of ecological interaction forged between producer and cross-feeder, we measured the net costs and benefits from association [32], [33] by comparing species growth rates when grown alone with their growth rates when grown in coculture (see Methods). If both species have an increase in growth rate relative to their growth rate in monoculture, the association is mutualistic. If both species have a decrease in growth rate when grown in coculture relative to their growth rate in monoculture, the association is competitive. If one species benefits at the expense of the other, then there is exploitation.

**Figure 1:**
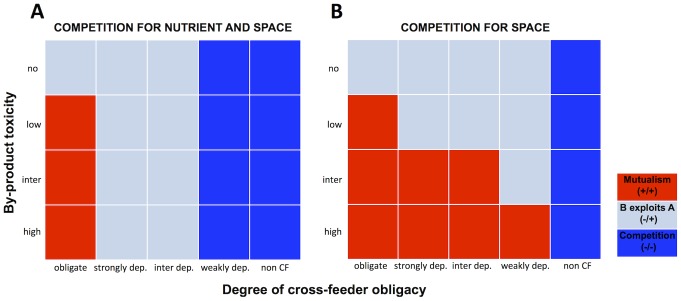
Metabolic interdependence dictates the ecological outcome of the food for detoxification interaction. Ecological outcome of interaction for varying by-product toxicity and degree of cross-feeder obligacy when the two species compete for both nutrients and space **A,** or compete for space only **B** (see Methods and Text for further details, and fig. S1 for a schematic representation of species interactions). Red indicates mutualism, gray indicates cross-feeder (B) exploits producer (A), and blue indicates competition. CF means cross-feeding.

Analytical work under the limiting assumption of a well-mixed (planktonic) community found that diverse ecological relationships can emerge from a one-way cross-feeding interaction where nutrients are traded for detoxification [25]. Does the same result hold when the environment is spatially structured? To address this question, we first investigated how the degree of metabolic interdependency (varying along two species axes) shapes the ecological relationships between two species. Specifically, we vary by-product toxicity from non-toxic to highly toxic (variations in metabolite toxicity can occur, for instance, via changes in pH or the type of metabolite produced [34]) and the degree of cross-feeder obligacy from non-cross-feeder to obligate cross-feeder (see fig. S1A for a schematic representation of species interactions, and Methods). Metabolic interdependency implies that a species' chemical environment is improved in at least one specific dimension by the presence of another species (for instance, detoxification or provision of a growth substrate). However this specific chemical aid does not imply that the recipient gains a net growth advantage from association, as the two species may also compete for other limiting resources (space and/or nutrients). We found that metabolic interdependency gives rise to diverse ecological interactions, ranging from mutualism to competition (figs. 1A, S2), thus corroborating our previous finding for well-mixed populations [25]. Specifically, mutualism only emerges when the specific help received outweighs the competitive costs endured for both partners.

In fig. 1A we have assumed that the two species compete for space and limited nutrients (unless cross-feeding is entirely obligate). We next ask what is the relative contribution of competition for space and nutrients to our results? Importantly, these two limiting resources are linked as winning the competition for space means getting access to nutrients, and similarly, winning the competition for nutrients means getting access to space. To disentangle their effects we relax nutrient competition (see schematic fig. S1B). As expected, we found that removing competition for nutrients leads to less negative associations, as seen by a shift from competition to exploitation, or from exploitation to mutualism (fig.1A, B). As toxicity increases, the ability of the producer to compete for a shared nutrient resource is diminished. Therefore, removal of nutrient competition has a disproportionately positive effect on mutualism as toxicity increases.

Our definition of mutualism ([25], [32]) implies that the total productivity of the two species community will be greater than the summed productivities of the two species apart. However, our results also show that enhanced community productivity does not itself imply mutualism, as exploitative relationships can also lead to a community gain (figs. S3, S4). This is consistent with empirical studies that have documented that resource (niche) partitioning via cross-feeding interactions enhances community productivity [35], [36], [37], [38], with the caveat that enhanced community productivity does not alone dictate a mutualistic relationship.

### Metabolic interdependence drives species intermixing

Theoretical modelling has suggested that population segregation (high relatedness) can favour within-species cooperation because segregation keeps the benefits of cooperation close to cooperators [21], [22], although these benefits are potentially mitigated by enhanced competition among kin [39], [40], [41]. Furthermore, it has been suggested that population mixing favours between-species cooperation because it facilitates the exchange of the benefits of cooperation, therefore creating a tension between within-species cooperation and between-species cooperation [22]. In our food for detoxification interaction, the effect of within-species cooperation on population segregation is relaxed, therefore allowing for between-species mutualism to occur under a broader range of conditions. In a recent simulation and experimental study, it has been shown that strong inter-population cooperation leads to inter-population mixing in microbial communities, and specifically in a pattern of successive layering [23].

**Figure 2:**
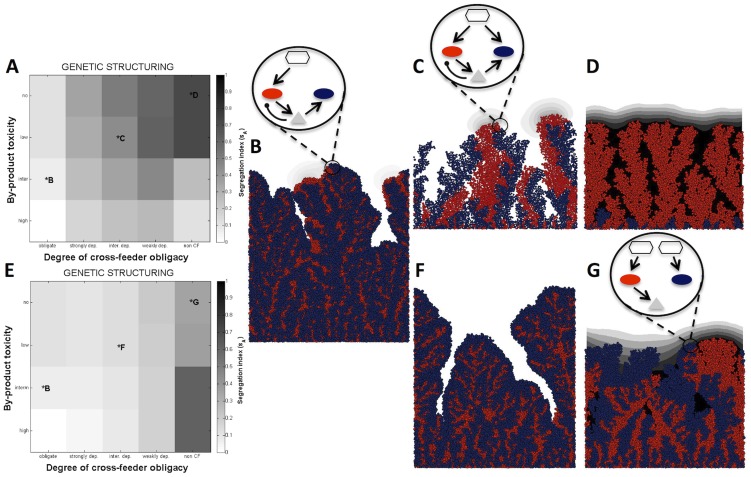
Metabolic interdependence drives genetic mixing. Producer segregation index (*s*_A_, see Methods) for varying by-product toxicity and degree of cross-feeder obligacy when the two species compete for both nutrients and space **A,** or compete for space only **E.** Lighter regions indicate greater mixing (see Methods for further details and fig. S5 for cross-feeder segregation index). Data are the mean of 3 replicates. **B–D, F, G.** Biofilm images of community growth from one of the associations represented in **A** or **E**. Producer is represented in red, and facultative cross-feeder, obligate cross-feeder, and non-cross-feeder are represented in blue. By-product is in gray. The schematics illustrate the metabolic interaction scenarios. Oval, hexagon, and triangle, represent bacteria, main nutrient, and by-product, respectively. Open arrows represent a positive effect, whereas oval arrows represent a negative effect upon the population or resource they are pointing toward. See fig. S1 for a complete schematic representation of all metabolic interaction scenarios.

Based on these observations, we next hypothesized that varying metabolic interdependence would dictate the degree of species intermixing within the two-species community, and in a way that reflects the net costs and benefits of interspecific association. In particular, we would expect that increasing metabolic interdependence would result in higher species intermixing within the biofilm to facilitate trade. We generally found that, as by-product toxicity increases, intermixing increases (figs. 2, S5). Similarly, increasing cross-feeder obligacy leads to higher intermixing (figs. 2, S5), except in the non-cross feeding medium (and intermediate to high by-product toxicity). The latter scenario likely occurs because the fast growing cross-feeder cells insulate the poorly growing producer cells in separate enclaves, thus leading to greater mixing. The segregation index (Methods) provides a global statistic of population structure, but does not reveal the developmental patterning of the two intermixing species or their resulting shared architecture. Videos S1 and S2 illustrate the resulting development and architecture of the two-species community, and highlight that strong mixing is achieved via a striking and emergent branching pattern producing increased inter-digitation and contact surface between interdependent cell lineages. Branching-like patterns within single clonal lineages have been observed previously under conditions of low nutrient availability, due to stochastic variations in a thin active growth layer [21]. The resulting separated ‘towers’ (observable in fig. 2D) are mutually repulsive, as growth towards conspecifics increases competition for limiting resources. In contrast, as mutual interdependence increases, demographic movement towards heterospecifics becomes increasingly rewarding, resulting in branching of lineages towards heterospecifics and away from conspecifics, generating a robust and stabilising mixing pattern.

### Strong interdependence generates more robust mixing

It has been recently documented that population intermixing of a yeast obligate cooperative community is robust to a broad range of initial conditions, including initial ratio and densities [23]. In this study, however, the authors assumed that cells were randomly seeded. Given this, we hypothesized that the degree of intermixing at inoculation may influence the ecological and structural relationship of the two species trading food for detoxification, by modulating the establishment of key metabolic and demographic feedbacks. Indeed, increasing segregation at inoculation might have two opposite effects: on the one hand, we would expect the costs of interspecific competition to be delayed, but on the other hand, the benefits of trade would be reduced.

To examine this, we repeated the simulations of monoculture, facultative cross-feeding coculture, and obligate cross-feeding coculture, but now the cells were inoculated in two microcolonies of size 30 µm and separated by a distance of 70 µm from each other (coculture) or in a single microcolony of size 30 µm (monoculture). The degree of initial intermixing was changed by varying the proportions of producer and cross-feeder in each microcolony but keeping the total number of inoculated cells constant and 1∶1. This means that, for example, when both microcolonies were inoculated with equal number of cells of producer and cross-feeder type, then they were completely intermixed (i.e. segregation index, *s*, equal to 0, see Methods). When one microcolony was inoculated with cells of producer type only and the other microcolony with cells of cross-feeder type only, then they were fully segregated (i.e. segregation index, *s*, equal to 1). Note that monoculture simulations were repeated using the same seeding rule to prevent any bias from inoculation crowding effects when we are comparing monoculture and coculture growth.

**Figure 3:**
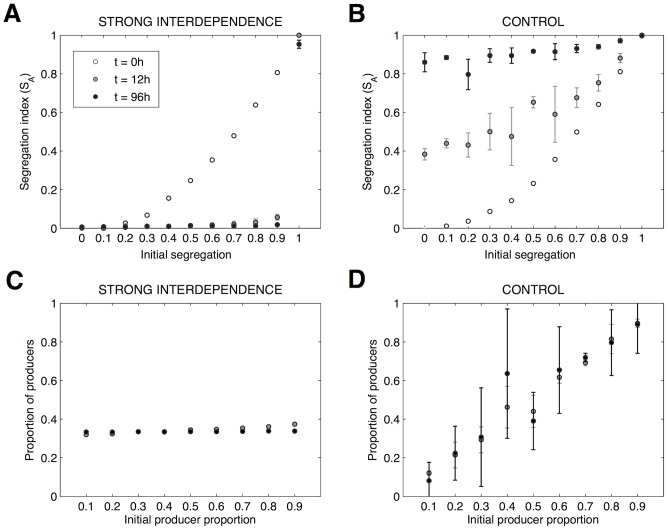
Strong interdependence generates communities that are robust to variation in initial conditions. **A, B,** Emergent population structure (segregation index, *s*_A_,) as a function of initial intermixing, for two scenarios. **A**, strong interdependence (i.e. obligate cross-feeding and high by-product toxicity) and **B**, no interdependence (control scenario). Population structure is recorded at inoculation (open circles), and after 12 (grey dots) and 96 (black dots) hours. Initial population structure was varied by varying the proportions of producer (species A) and cross-feeder cells (species B) in two adjacent micro-colonies (of size 30 µm separated by a distance of 70 µm) while maintaining a constant total inoculation density and ratio (1∶1). An initial segregation 0 means that each microcolony received equal numbers of A and B, whereas initial segregation of 1 means that one microcolony was pure A and the other pure B. An increment in initial segregation of 0.1 means a 5% increase (or decrease) in the number of cells of species A (or species B) inoculated in each microcolony. **C, D.** Proportion of producers as a function of initial producer proportion for strong interdependence (i.e. obligate cross-feeding and high by-product toxicity) and control scenario, respectively, and after 12 (grey dots) and 96 (black dots) hours growth (initial segregation = 0). Data are the mean of 3 replicates and error bars are the SD of the mean.

In the absence of metabolic interaction, the two species (here, differing only in colour) tend to segregate, independently of initial intermixing (fig. 3B). This agrees with modelling [21] and empirical work on the social amoeba *Dictyostelium discoideum* [42] showing that spatially structured growth is a passive mechanism that increases relatedness (or lineage segregation). But what happens when the lineages experience metabolic interactions? Our results suggest that the emergent patterns of lineage mixing (fig. 2A) are highly robust against variation in initial inoculum mixing, except when the two species are completely segregated in two separate microcolonies at inoculation (fig. 3A, fig. S6A–C). Indeed, if the two species are strongly interdependent, they are conditioned to mix to grow. Thus, when initially segregated, such strong initial segregation may delay (fig. S7A, B) or even prevent (e.g. when interdependency is too high; fig. S7C) the structural relationship to be forged. This result also supports the idea that spatial distance between species plays a critical role for the stable coexistence of obligate mutualistic bacterial communities [19]. We also found that the strongly interdependent community shows a strong signature of negative frequency dependent selection (the rare lineage is favoured), ensuring a stable coexistence frequency of around 34% producers, regardless of their initial frequency (fig. 3C). In contrast, the control community is sensitive to the proportion of producers at inoculation (fig. 3D), due to the absence of stabilising mechanisms of interaction.

### Demographic drivers of intermixing

**Figure 4:**
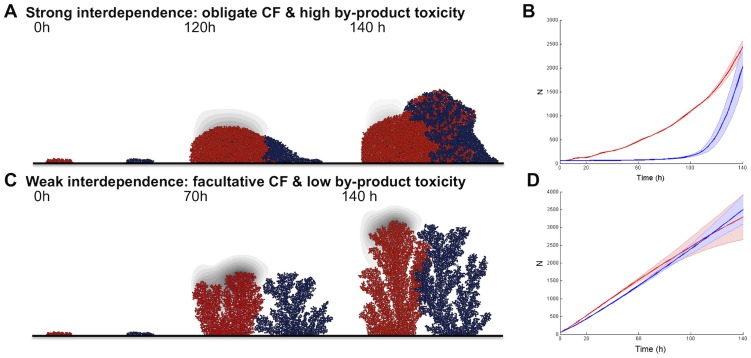
Demographic signatures of functional relationships given initial species segregation. **A, B.** The two species are strongly interdependent. **C, D.** The two species are weakly interdependent. Producer is represented in red and cross-feeder is represented in blue. By-product is in gray. Simulations were initiated with two segregated microcolonies (1∶1). Boundaries on the sides are permeable to the by-product and non-cyclic. **B, D.** Time series of species biomass (N). The thick lines represent the mean (n = 9) and shaded areas represent the standard deviation.

To further understand the demographic drivers of intermixing, we break the demographic feedbacks by modifying both initial segregation conditions and the mass-transfer regime (by-product diffusion). First, we simulated the growth of an initially segregated two-species community and separately tracked the growth rates of cells situated nearer towards or further apart from the heterospecific lineage. We found that when metabolic interdependence is high, the cells that are closer to interspecific cells grow better than the cells that are further away from interspecific cells (fig. S8A). As shown in Video S3, obligate cross-feeder cells closer to producer cells grow towards the producer cells, i.e. towards the by-product. In turn, this reduces the concentration of toxic by-product in the microenvironment of producer cells that are closer to the obligate cross-feeder, thus favouring the growth of those neighbouring producer cells. This result highlights the importance of demographic feedbacks that follow from growth benefits of trading resources for detoxification in shaping community function and spatial structure. At a more macroscale, the results of demographic feedbacks among mutualists are clear in fig. 3C, where we see the signature of negative frequency-dependence driving the two partners to a stable coexistence point irrespective of initial frequency, and in fig. 4AB where we see an accelerating growth of mutualists with increasing heterospecific proximity and mixing.

Furthermore, as observed earlier for intermixed inocula (fig. 2B and Video S1), the community branching structure emerges as the community grows, but the branching pattern is now- with separated inocula- more pronounced, probably because of reduced space constraints (fig. 4A, Video S3). The emergence of similar architectures and intermixing statistics between Videos S3 and S1 (i.e. separate and intermixed inocula, respectively) highlights the robust community developmental programme that results from strong metabolic interdependencies, which in turn deliver a high-functioning community.

Given facultative cross-feeding, the cross-feeder can grow using the shared limiting nutrient (e.g. glucose) as well as the producer by-product. When the by-product is weakly toxic both producer and cross-feeder cells that grow closer to interspecific cells grow better than the cells that are further away (fig. S8B, Video S4), but the disadvantage of cells growing further away is now smaller and mixing is reduced. As by-product toxicity increases, producer cells growing closer to the cross-feeder can even grow more slowly than those further away, despite receiving greater detoxification benefits. The producer cells adjacent to cross-feeding cells suffer due to the increased competition for the shared limiting nutrient (fig. S8B). At a more macroscale, the results of demographic feedbacks among weakly interdependent partners (fig. 4CD) can be seen by a negative correlation between the densities of producer and cross-feeder across replicates following lineage contact (fig. S9B), as a stochastic advantage to one lineage spells a cost to the competitor lineage (generating the increased variance around the mean in fig. 4D). In contrast, strong interdependence generates a positive correlation between producer and cross-feeder across replicates following contact (fig. S9A), as a stochastic advantage to one lineage drives further advantages to its partner lineage.

**Figure 5:**
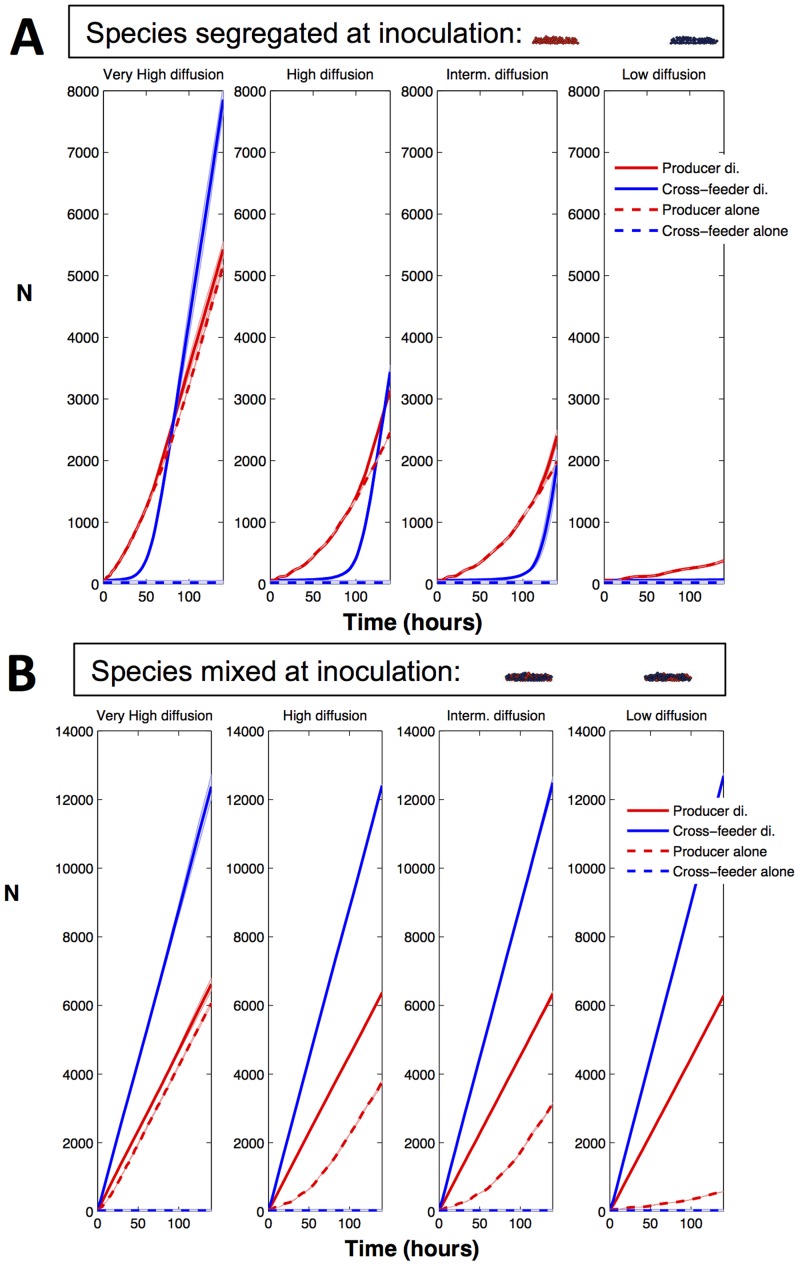
Effect of varying diffusion and initial segregation on the emergent properties of strongly interdependent communities. **A,** The two species are initially segregated. **B,** The two species are initially mixed. Time series of species biomass (N) when grown in diculture (solid line) or alone (dashed line). The thick lines represent the mean (n = 3) and shaded areas represent the standard deviation. See fig. 3 legend for further details on seeding conditions. By-product diffusion rates are [10*D_E_*; 1.4*D_E_*; *D_E_*; 0.14*D_E_*] from very high to low, respectively (see Table S2).

The ability to effectively carry out a food-for-detoxification exchange depends ultimately on an effective process of molecular transport from producer to consumer cell. In our final manipulation, we vary the rate of diffusion to explore the importance of mass-transfer processes on the establishment and maintenance of metabolic and demographic feedbacks. We found that when the two species are initially spatially segregated, increasing diffusion improves the performance of both species, due to an enhanced metabolic flux kick-starting the exchange (figs. 5A, S10A). In contrast, when the two species are initially mixed, performances (lineage growth rates) are scarcely touched by changes in diffusion over two orders of magnitude, as the initial proximity of the partner lineages assures effective inter-cellular transport even at very low rates of diffusion (figs. 5B, S10B). The effect of diffusion is however very pronounced on the resulting strength of mutualism. When diffusion is very low, mutualism is far stronger simply because the producers are in much more trouble when alone (fig. 5B). In contrast, as diffusion increases, solitary producer colonies suffer less from their byproduct toxicity due to a rapid abiotic removal process, making the net benefit of partnership much weaker (fig. 5B). Together, these results illustrate the important and interacting roles played by initial segregation and diffusion in establishing an effective metabolic exchange, and consequently the emergent function and spatial structure of communities.

## Discussion

While it is well acknowledged that spatial structure plays a critical role in shaping the ecological outcome of species interactions, our understanding of how community structure and function emerge from the mechanistic bases of species interactions is still poorly understood. Here, we addressed this question by investigating how metabolic constraints of individual species shape the emergent functional and structural relationships of a two-species microbial community that trades food for detoxification.

Specifically, our main findings reveal that mutual interdependence generates a robust and highly stabilising mixing pattern. This happens because demographic movement towards heterospecifics becomes increasingly rewarding, resulting in branching of lineages towards heterospecifics and away from conspecifics. These demographic feedbacks strengthen mutualistic relationships via increased lineage mixing, and weaken competitive relationships via increased segregation. Furthermore, we show that initial mixing and diffusion play a critical role in establishing effective metabolic exchange, and therefore in defining the emergent functional and structural relationships among species.

Strong metabolic interdependence is commonly found in syntrophic (cross-feeding) relationships [43], and empirical evidence for the importance of spatial distribution in the functioning of metabolically interdependent syntrophic consortia is growing in the literature [44], [45]. But, what if mutualism is based on bidirectional cross-feeding rather than a food for detoxification mutualism? Recent work has suggested that strong inter-population cooperation, in which each strain depends on the provision of an essential metabolite by the other strain, leads to population mixing in a pattern of successive layering (for yeast see [23], for *E. coli* see [46]). One potential explanation for this discrepancy in spatial pattern between their findings and ours is the specific nature of the mechanistic interaction (e.g. bidirectional cross-feeding *vs* food for detoxification cross-feeding). To assess this possibility we ran additional simulations assuming bidirectional cross-feeding instead of food for detoxification cross-feeding and we observed a hybrid result. We still observe a characteristic emergent branching pattern, although now the producer forms a mantle layer at the top of the biofilm (fig. S11). Understanding the drivers of these distinct spatial patterns is an interesting area of research to be pursued.

A striking result in our simulations is the emergent two-species branching structure of communities that exhibit strong interdependence (Videos S1, S3). Branching patterns are commonly found in nature (e.g. neurons, blood vessels, trees). In bacteria, branching has been observed in swarming colonies, including *Bacillus subtilis* [47] and *Pseudomonas aeruginosa* [48], [49], [50], but what may explain such community architecture here? Branching seems to emerge because of lineage growth with demographic movement away from conspecifics and towards interspecifics (helpers), thereby maximizing the surface contact area with interspecifics. Video S3 suggests that the first mover is the obligate cross-feeder which branches into regions of high by-product concentrations (high toxicity for producer). This relieves inhibition on the producer, which can now grow until toxicity returns.

Here, we have assumed that the facultative cross-feeder is able to use both the common resource and the by-product independently of their concentrations in the environment. This means that the trade-off between the cross-feeder's ability to use both nutrients is fixed, and not under regulatory control. Regulatory control, however, plays a critical role in bacterial metabolism and social dynamics [48], [51]. One could relax this assumption and allow for regulatory control in our cross-feeding model. For example, common resource vs by-product consumption could be a plastic trait that depends on the local concentration of the by-product. Specifically, one could assume a scenario where the metabolism of the by-product inhibits the uptake of the common resource [52]. While outside the scope of this study, we believe that investigating how metabolic plasticity in resource use affects the structure-function dynamics of interspecific interactions would add to our understanding of mapping metabolism to ecology and structure in polymicrobial communities.

Our work looks at interspecific mutualisms that arise due to by-product mutualisms, as the benefit provided to the other species occurs as a result of a trait carrying no immediate, direct cost to the actor [33], [53]. Additionally, our model assumes that cell movement is purely due to demographic processes of cell growth. This means that there is no behavioural mechanism that preferentially directs help towards a mutualistic partner (such as in partner choice, [13], [53]) or makes an individual preferentially move towards a mutualistic partner. While it is unclear whether partner choice exists in bacteria, motility [54] and chemotaxis are behavioural mechanisms that allow bacterial cells to move towards favourable environments (e.g. food gradient) and therefore influence species functional and structural relationships. It would be interesting to see how these mechanisms would affect the functioning and structuring of our food for detoxification association. One would nevertheless expect a similar general structural pattern even when behavioural processes are at play, i.e. mix when the benefits of association outweigh the costs, but segregate when the costs of association outweigh the benefits.

Another explicit assumption of our model is that cells are growing on an inert surface and that the nutrient diffuses from the bulk (above) into the biofilm. This implies that only the cells that are at the surface of the biofilm are able to access the nutrient and grow. This is a common assumption when using this individual-based framework, but this may not always be the case as in, for example, the gut environment (see [27] for an individual-based model of host-microbiota interactions where the authors assume bidirectional nutrient gradient). Under these conditions, and assuming sloughing of microbial cells, different emergent structures and branching patterns may arise.

Our study illustrates how community structural and functional relationships emerge from metabolic signatures of interspecific interaction. Although we focused on a specific mechanism of trade - food for detoxification of a metabolic by-product - we believe that our approach of mapping metabolism into function and spatial organization can be extended to other types of microbial associations. It would be interesting, for instance, to investigate what are the emergent functional and structural relationships of a two-species community trading food for detoxification of an exogenous toxic metabolite (e.g. antibiotic). Also, trading food for detoxification implies that when mutualism emerges, it is intrinsically resistant to interspecific cheating strategies. This conclusion lends greater relevance to our ecological results, however it still leaves open a number of questions on the potential for coevolutionary dynamics within this mutualistic space, for instance towards greater rates of waste production [25].

Finally, we suggest that further research into the interplay between the molecular mechanisms of species interactions and the ensuing population and community dynamics is needed to foster our understanding of how natural microbial communities emerge and are maintained in the first place, as well as predict how they may be affected by environmental perturbations on both ecological and evolutionary timescales [55].

## Methods

### Model

Our model assumes two species, a producer (A) of a metabolic by-product (E), and a cross-feeder (B) (see fig. S1 for a schematic representation) growing on an inert surface. The producer and cross-feeder are ecological competitors for a common limiting nutrient (R, e.g. glucose) that diffuses from the bulk (above) into the biofilm. The bulk consists of a liquid and well-mixed compartment where the concentration of nutrient (R_bulk_) is held constant. Thus, the growths of species A, and species B, are a function of the rates of uptake of R in the local microenvironment of A and B, respectively. In addition, the cross-feeder's growth is enhanced by its ability to use the producer waste product E, while the producer's growth is decreased by E (i.e. toxic waste product). Thus the concentrations of R and E vary in space and time due to production/consumption reactions and diffusion.

The metabolic reactions and stoichiometric matrix used in the model are described in detail in Table S1. Briefly, the reaction of transformation of R into E and biomass A (X_A_, cell growth of A) follows a Monod-form kinetic, and E inhibits this reaction via simple inhibition. The reaction of transformation of R and E into biomass B (X_B_, cell growth of B) follows a Monod-form kinetic on R and E, respectively. Also, we assume that the producer has more affinity and is more efficient at using the main nutrient (R) than the cross-feeder, such that *K_R, A_<K_R, B_* and *Y_R, A_>Y_R, B_*, respectively. This may represent, for example, a cost of resource generalism to the cross-feeder [56]. We assume that the obligate cross-feeder (B_obl_) is specialist on the producer's waste product and incapable of using the limiting nutrient. This means that the two species do not use overlapping nutrients and that the cross-feeder depends on its partner's waste product for growth. Specialization on a partner's waste product of metabolism can occur via mutations [57] or due to an exclusion mechanism in which the metabolism of the waste product inhibits the uptake of the limiting nutrient [52]. In addition, we assumed three facultative cross-feeders. Consistent with previous empirical work we assume that the facultative cross-feeders are able to use both the common limiting nutrient and the metabolic by-product (see e.g. [36], [37], [57], [58]), but differ in their degree of obligacy, varying from strongly dependent (B_facS_) to intermediately dependent (B_facI_) to weakly dependent (B_facW_) on the producer's waste product for growth (see Table S2). Finally, in the producer- non-cross-feeder (B_ncf_) association there is complete overlap of resource use. Specific parameter values used for the simulations are described in Table S2, and other simulation parameter values used for the simulations are described in previous work [22]. Unless otherwise stated, we assume cyclic boundary conditions.

Inoculation densities are 60 cells in monoculture, and 60 cells of each species (1∶1) in coculture. This means that the initial density of each individual species is held constant across culture type (i.e. monoculture and coculture), and thereby the total inoculation density of monoculture is half the total inoculation density of coculture (additive experimental design). This approach gives us a measure of how an individual species is affected by diversity only, and not by initial individual species densities.

### Measuring growth rate

Growth rate is measured as (*N_f_*−*N_i_*)/(*t_f_*−*t_i_*) where *N_i_* represents the number of cells inoculated at time 0 (*t_i_*), and *N_f_* represents the number of cells at the end of the simulation (*t_f_*). Unless otherwise stated, data represent growth after 96 hours, and are the mean of 3 replicates.

### Segregation index

The segregation index (*s*) is an indicator of species segregation (or mixing) within their local neighbourood measured relative to global species frequencies, and is measured as:
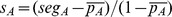
and
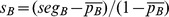
where *seg_A_* (*seg_B_*) represents the proportion of species A (species B) in the local microenvironment (i.e. neighbourhood), and 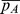 (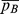) is the proportion of species A (species B) in the whole population. Note that this way of measuring species segregation in an interspecific population is similar to the relatedness coefficient used in social evolution to measure relatedness within-species [42], [59]. This intermixing index can also be seen as an indicator of species co-assortment, e.g., whether species A is more assorted (or segregated) with species B than if the two species were distributed randomly (i.e. when *s* = 0).

The calculation of the proportions of species A and species B in the local environment is adapted from the methodology used in Mitri et al. [22] to measure population segregation in biofilm. In brief, for each individual cell (*c_i_*) of a given species - i.e. either species A or species B- in a population of *N* = *N_A_*+*N_B_* cells we identify all the neighbour cells (*c_j_*) falling within a neighbourhood distance of a radius of 10 µm. The segregation of each individual cell *c_i_* is defined as:
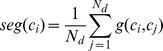
where *g*(*c_i_*, *c_j_*) = 0 if *c_i_* and *c_j_* belong to different species, or, *g*(*c_i_*, *c_j_*) = 1 if *c_i_* and *c_j_* belong to the same species, and *N_d_* is the number of cells falling within the distance of 10 µm. The segregation index *seg_A_* (*seg_B_*) of species A (species B) is then defined as:
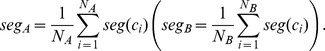



## Supporting Information

Figure S1 **Schematic representation of species interactions.** **A,** The two species compete for a common nutrient and space. From left to right: Obligate food for detoxification, i.e. no competition for the shared nutrient; Facultative food for detoxification, i.e. the cross-feeder is able to use both by-product and common nutrient; Non cross-feeding medium, i.e. complete overlap in resource use and no cross-feeding; and, control community where both species are identical except for their color (see text for more details). **B,** The two species compete only for space. Oval, hexagon, and triangle, represent bacteria, main nutrient, and by-product, respectively. Open arrows represent a positive effect, whereas oval arrows represent a negative effect upon the population or resource they are pointing toward.
(TIFF)

Figure S2 **Benefits of association increase with need for help (need for detoxification, and need for food).** **A, B.** Data represent log growth rate of producer (cross-feeder) in coculture relative to producer (cross-feeder) in monoculture for varying by-product toxicity and cross-feeder degree of obligacy. Measured as log(*X*_co_/*X*_mono_) where *X*_co_ and *X*_mono_ represent growth rate in coculture and monoculture, respectively (for growth rate calculation see Methods). To note that obligate cross-feeder growth rate is measured as log(*B_co_*) because the obligate cross-feeder cannot grow in monoculture. Positive and negative values indicate a net gain and loss from association, respectively.
(TIFF)

Figure S3 **Enhanced community productivity does not itself imply mutualism.** **A,** The two species compete for a common nutrient and space. **B,** The two species compete only for space. Indeed, exploitative relationships can also lead to a community gain (see fig. 1). Data represent (A_co_+B_co_)−(A_mono_+B_mono_) and are the mean of 3 replicates. The black line separates the gain (+) and loss (−) regions.
(TIF)

Figure S4 **Metabolic interdependence drives community functioning (productivity).** **A.** Productivity of the community (A_co_+B_co_), and **B.** sum of monocultures (A_mono_+B_mono_) for varying by-product toxicity and degree of cross-feeder obligacy (see Methods for further details). Data are the mean of 3 replicates.
(TIF)

Figure S5 **Genetic mixing increases with need for help (need for detoxification, and need for food).** Cross-feeder segregation index (*s*_B_) for varying by-product toxicity and degree of cross-feeder obligacy (see Methods section for further details). Lighter regions indicate greater mixing. Data are the mean of 3 replicates.
(TIFF)

Figure S6 **Stronger interdependence generates more robust community intermixing to intermixing at inoculation.** **A–C.** Obligate cross-feeding (A−B_obl_). **D–F.** Facultative cross-feeding (A−B_facI_ scenario). Two microcolonies of size 30 µm separated by a distance of 70 µm were inoculated with varying proportions of producer and cross-feeder cells but constant inoculation density (1∶1). In the x-axis, 0 means that the two microcolonies were inoculated with equal number of cells of species A and B and represents *s*∼0, whereas 1 means clonal microcolonies at inoculation, and therefore *s* = 1. An increment of 0.1 means a 5% increase (or decrease) in the number of cells of species A (or species B) inoculated in each microcolony. Data represent producer segregation index at inoculation (white circles), and after 12 and 96 hours growth (grey and black dots, respectively). Data are the mean of 3 replicates and error bars are the SD of the mean. **A, D,** low by-product toxicity; **B, E,** intermediate by-product toxicity; **C, F,** high by-product toxicity.
(TIFF)

Figure S7 **Costs and benefits of association for varying degree of intermixing at inoculation.** **A–C, G–I.** Obligate cross-feeding, after 12 h and 96 h growth, respectively. **D–F, J–L.** Facultative cross-feeding (A−B_facI_) after 12 h and 96 h growth, respectively. **M–N.** Control, after 12 h and 96 h growth, respectively. Measured as log(*X*_co_/*X*_mono_) where *X*_co_ and *X*_mono_ represent growth rate in coculture and monoculture, respectively (for growth rate calculation see Methods). To note that obligate cross-feeder growth rate is measured as log(*B_co_*) because the obligate cross-feeder cannot grow in monoculture. Positive and negative values indicate a net gain and loss from association, respectively. Red dots represent producer, blue squares represent obligate cross-feeder, and blue dots represent facultative cross-feeder. In the control scenario, the two types are identical except for their color, i.e. red-tagged cells or blue-tagged cells. See legend fig. 3 for details on inoculation conditions.
(TIFF)

Figure S8 **Effect of interspecific partner proximity at seeding.** **A.** Obligate cross-feeding. **B,** Facultative cross-feeding (A−B_facI_). Growth rate advantage is measured as the difference between the growth rate of a producer (cross-feeder) growing close to a cross-feeder (producer) and the growth rate of a producer (cross-feeder) growing far from a cross-feeder (producer). Thus, positive values mean a growth rate advantage from interspecific partner proximity whereas negative values mean a growth rate disadvantage from interspecific partner proximity. Boundaries on the sides of the domain are permeable to the by-product and non cyclic. Data represent 120 hours growth, are the mean of 3 replicates, and error bars are the SD of the mean.
(TIFF)

Figure S9 **Results of demographic feedbacks on functional relationships.** **A.** The results of demographic feedbacks among strongly interdependent partners can be seen by a positive correlation between the densities of producer and cross-feeder across replicates following lineage contact (fig. 4AB). **B.** The results of demographic feedbacks among weakly interdependent partners can be seen by a negative correlation between the densities of producer and cross-feeder across replicates following lineage contact (fig. 4CD). See legend fig. 4 for further details.
(EPS)

Figure S10 **Effect of by-product diffusion rate on strongly interdependent communities given initial segregation A, and, initial mixing B.** Producer segregation index (s_A_*) was measured for a neighbourhood of 5 um (see legend fig. 3 and Methods section for further details). Given the strong mixing pattern of strongly interdependent communities, here we decreased the size of the neighbourhood to measure spatial structuring even more locally. By-product diffusion rates are [10*D_E_*; 1.4*D_E_*; *D_E_*; 0.14*D_E_*] from very high to low, respectively (see Table S2). Data are the mean of 3 replicates.
(TIFF)

Figure S11 **Emergent branching pattern of a two-species community involved in bidirectional nutritional benefits (cross-feeding).** The schematic illustrates the metabolic interaction scenario. Specifically, the two species are identical in their cross-feeding capabilities but species A (red) is also able to use the limiting nutrient (hexagon) while species B (blue) is obligate on species A's by-product for growth. By-products are represented by triangles. Biofilm image after 60 h growth.
(TIFF)

Table S1 **Reactions and respective stoichiometry of biological processes used in the models.**
(PDF)

Table S2 **Model parameters.**
(PDF)

Video S1 **Simulation of the producer-obligate cross-feeder community growth represented in** **fig. 2B** **.** This simulation shows that stronger interdependency leads to greater mixing and illustrates the emergent branching pattern.
(MOV)

Video S2 **Simulation of the producer-facultative cross-feeder community growth represented in** **fig. 2C** **.** This simulation illustrates that weaker interdependency leads to lower mixing. Some degree of community branching is observed.
(MOV)

Video S3 **Simulation of the producer-obligate cross-feeder community growth illustrating that when metabolic interdependence is strong the cells that are closer to interspecific cells grow better than the cells that are further away from interspecific cells.** Intermediate by-product toxicity scenario. Initial conditions: two clonal microcolonies were seeded 1∶1 with either producer (red) or obligate cross-feeder cells (blue). Light red and dark red cells were seeded 1∶1 on the left and right side, respectively, of the producer microcolony. Dark blue and light blue cells were seeded 1∶1 on the left and right side, respectively, of the cross-feeder microcolony. Boundaries on the sides of the domain are permeable to the by-product and non cyclic.
(MOV)

Video S4 **Simulation of the producer-facultative cross-feeder community growth illustrating that the cells that are closer to interspecific cells grow better than the cells that are further away from the interspecific cells.** However, given the weaker interdependence the cells growing further away from their interspecific partner grow better than when strongly interdependent. Mixing is thus reduced. Low by-product toxicity scenario. Initial conditions: two clonal microcolonies were seeded 1∶1 with either producer (red) or facultative cross-feeder cells (blue). Light red and dark red cells were seeded 1∶1 on the left and right side, respectively, of the producer microcolony. Dark blue and light blue cells were seeded 1∶1 on the left and right side, respectively, of the cross-feeder microcolony. Boundaries on the sides of the domain are permeable to the by-product and non cyclic.
(MOV)
