# Supplementary figures and images for: Metabolic and Demographic Feedbacks Shape the Emergent Spatial Structure and Function of Microbial Communities
Source: PLoS Comput Biol. 2013 Dec 26;9(12):e1003398. doi: 10.1371/journal.pcbi.1003398 (PMC3873226; doi:10.1371/journal.pcbi.1003398)

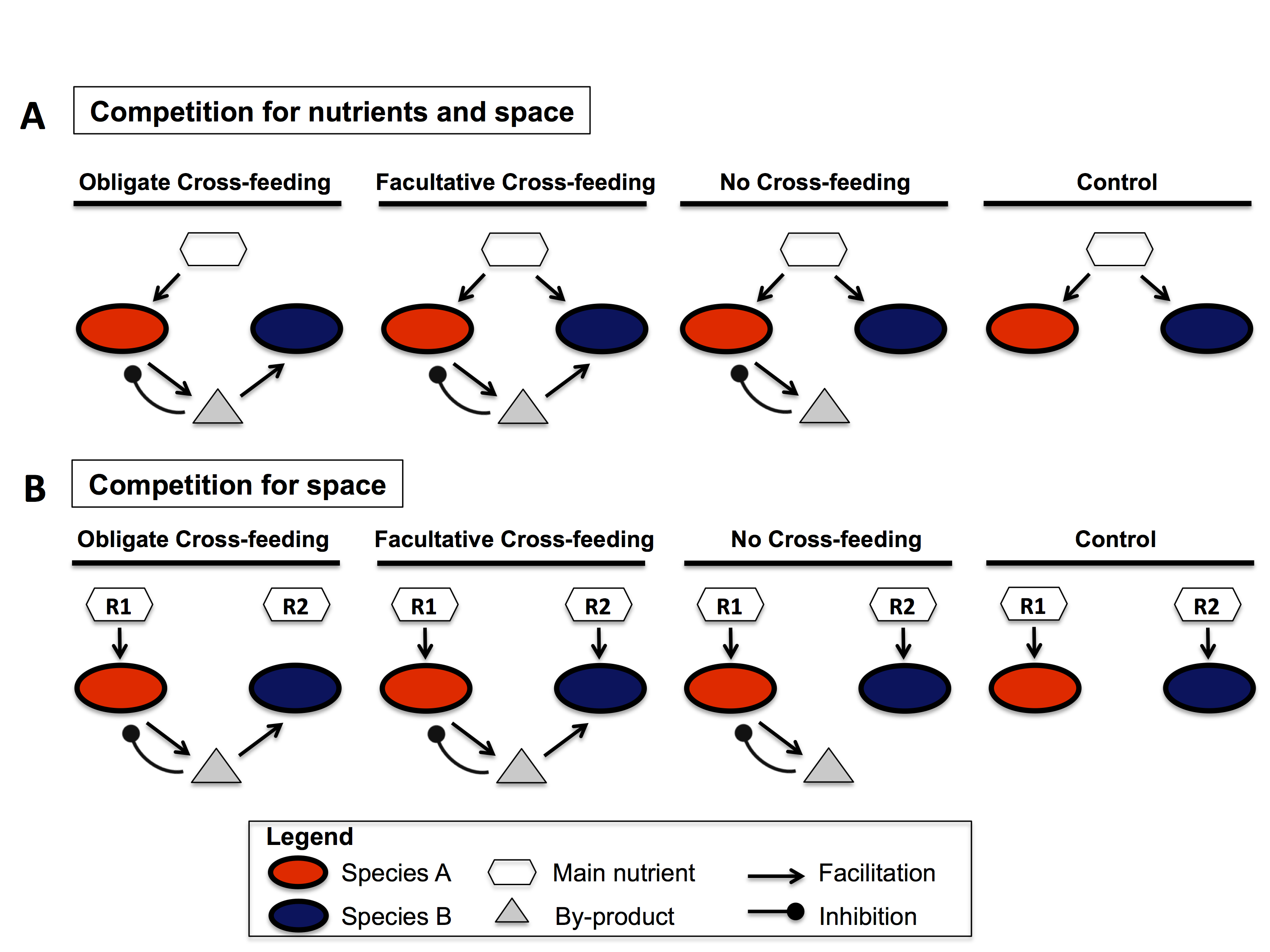

Supplement: Figure S1 — Schematic representation of species interactions. A, The two species compete for a common nutrient and space. From left to right: Obligate food for detoxification, i.e. no competition for the shared nutrient; Facultative food for detoxification, i.e. the cross-feeder is able to use both by-product and common nutrient; Non cross-feeding medium, i.e. complete overlap in resource use and no cross-feeding; and, control community where both species are identical except for their color (see text for more details). B, The two species compete only for space. Oval, hexagon, and triangle, represent bacteria, main nutrient, and by-product, respectively. Open arrows represent a positive effect, whereas oval arrows represent a negative effect upon the population or resource they are pointing toward. (TIFF) [file pcbi.1003398.s001.tiff]

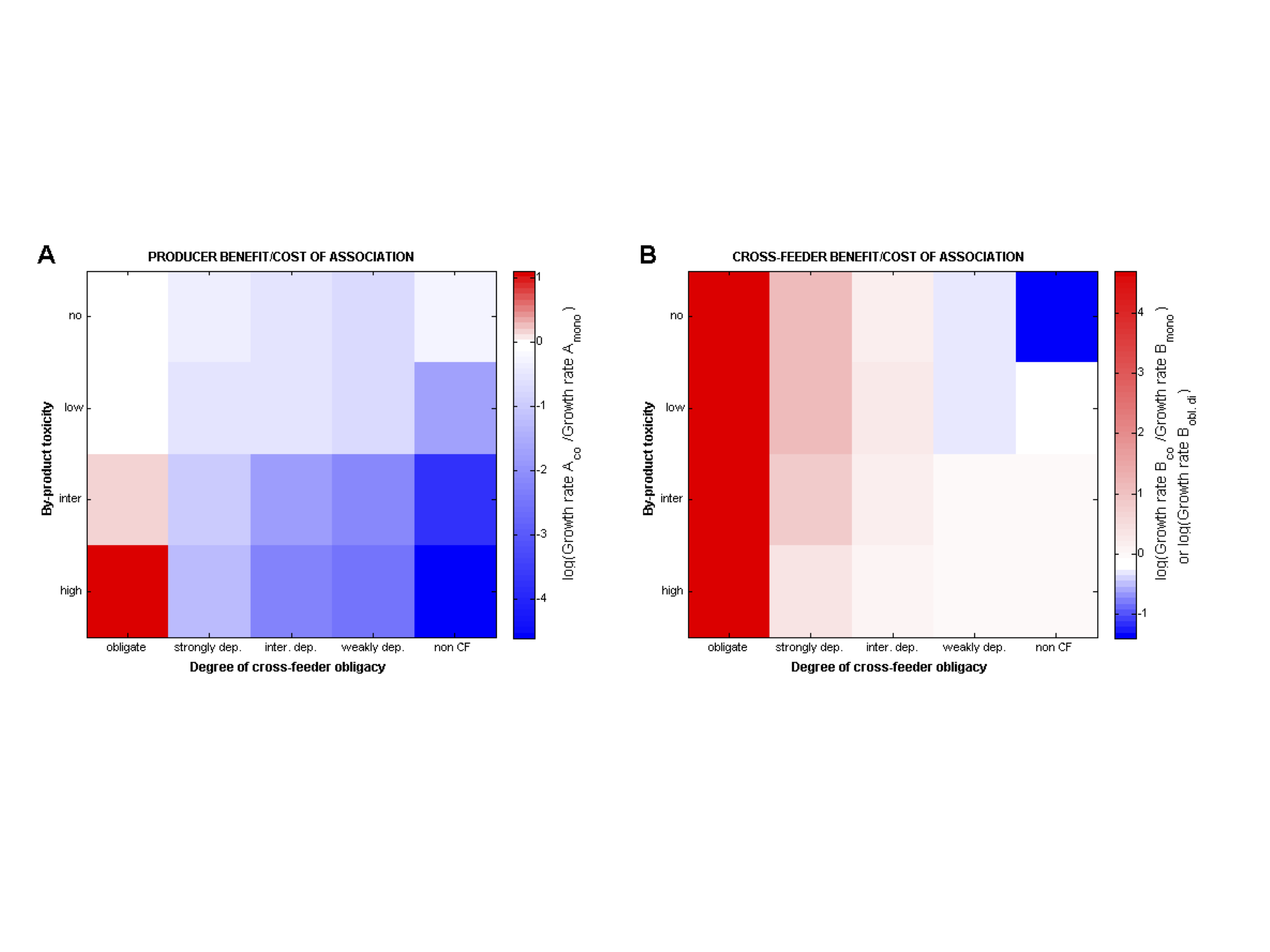

Supplement: Figure S2 — Benefits of association increase with need for help (need for detoxification, and need for food). A, B. Data represent log growth rate of producer (cross-feeder) in coculture relative to producer (cross-feeder) in monoculture for varying by-product toxicity and cross-feeder degree of obligacy. Measured as log(X co/X mono) where X co and X mono represent growth rate in coculture and monoculture, respectively (for growth rate calculation see Methods). To note that obligate cross-feeder growth rate is measured as log(Bco) because the obligate cross-feeder cannot grow in monoculture. Positive and negative values indicate a net gain and loss from association, respectively. (TIFF) [file pcbi.1003398.s002.tiff]

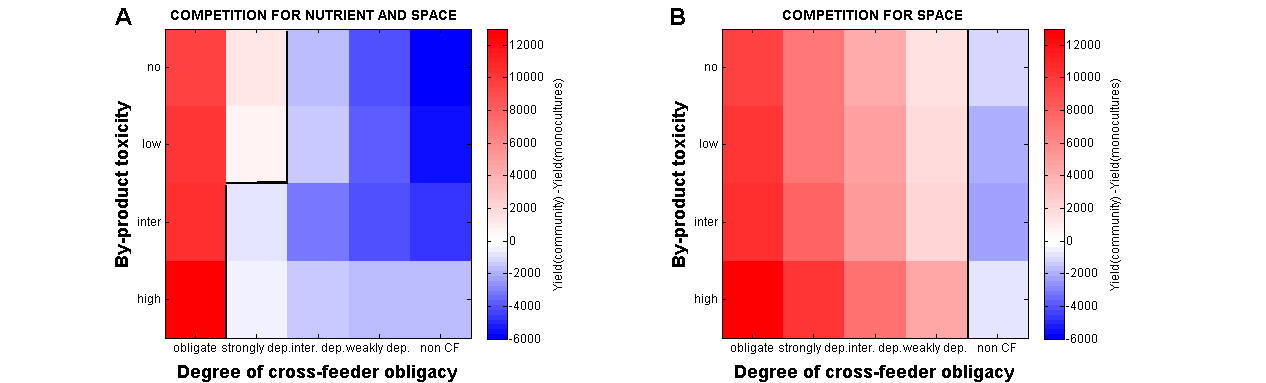

Supplement: Figure S3 — Enhanced community productivity does not itself imply mutualism. A, The two species compete for a common nutrient and space. B, The two species compete only for space. Indeed, exploitative relationships can also lead to a community gain (see fig. 1). Data represent (Aco+Bco)−(Amono+Bmono) and are the mean of 3 replicates. The black line separates the gain (+) and loss (−) regions. (TIF) [file pcbi.1003398.s003.tif]

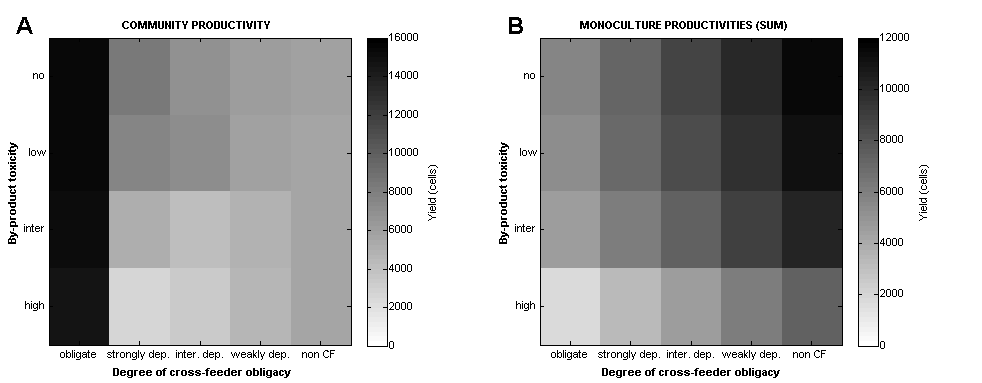

Supplement: Figure S4 — Metabolic interdependence drives community functioning (productivity). A. Productivity of the community (Aco+Bco), and B. sum of monocultures (Amono+Bmono) for varying by-product toxicity and degree of cross-feeder obligacy (see Methods for further details). Data are the mean of 3 replicates. (TIF) [file pcbi.1003398.s004.tiff]

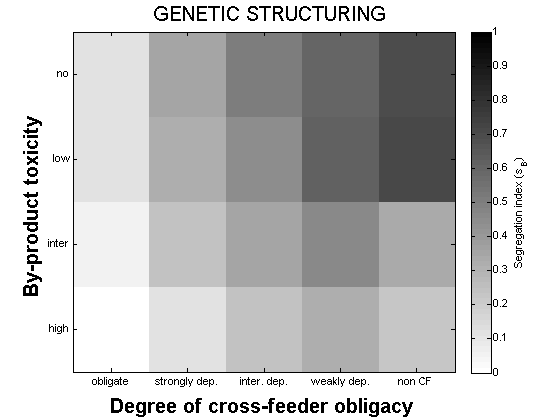

Supplement: Figure S5 — Genetic mixing increases with need for help (need for detoxification, and need for food). Cross-feeder segregation index (s B) for varying by-product toxicity and degree of cross-feeder obligacy (see Methods section for further details). Lighter regions indicate greater mixing. Data are the mean of 3 replicates. (TIFF) [file pcbi.1003398.s005.tif]

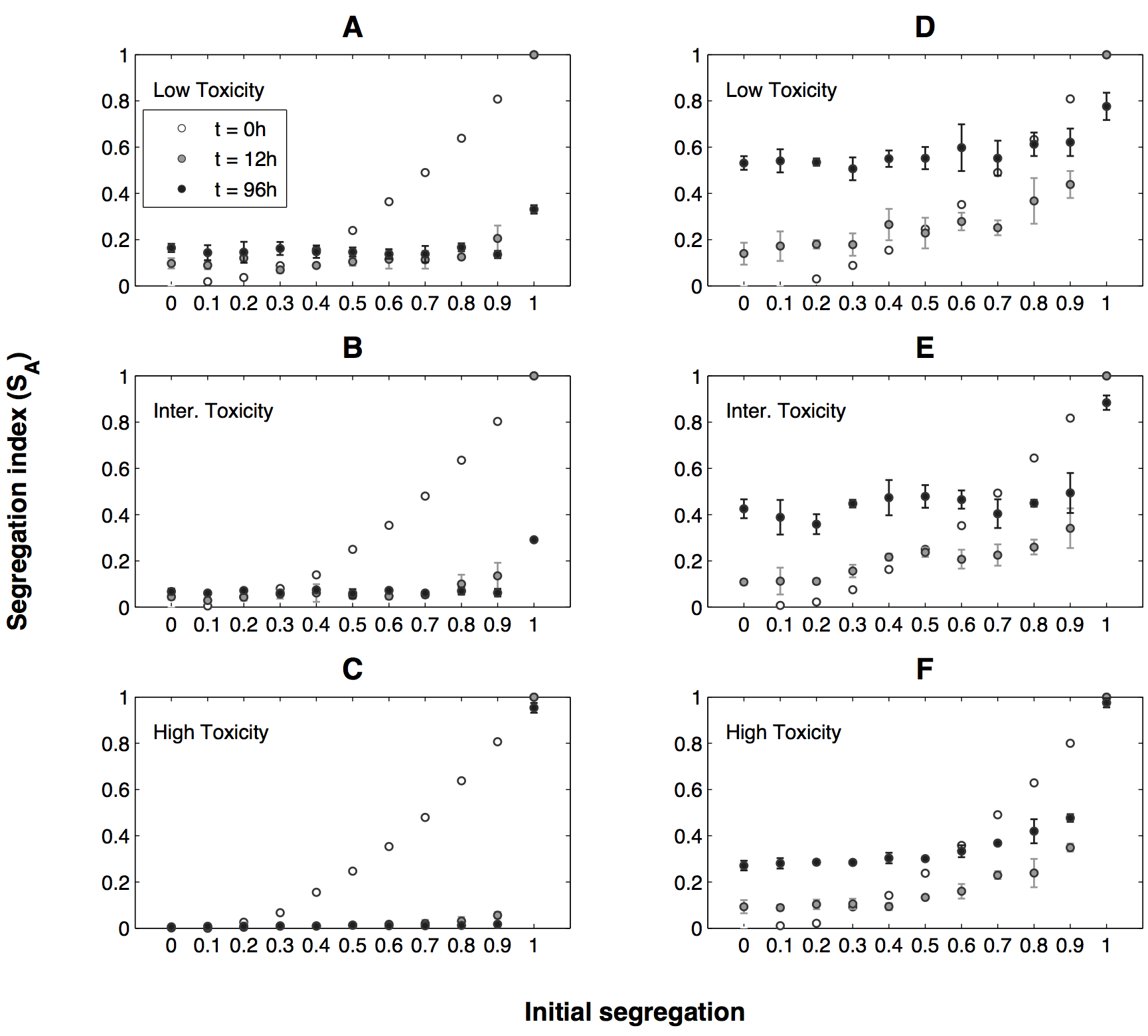

Supplement: Figure S6 — Stronger interdependence generates more robust community intermixing to intermixing at inoculation. A–C. Obligate cross-feeding (A−Bobl). D–F. Facultative cross-feeding (A−BfacI scenario). Two microcolonies of size 30 µm separated by a distance of 70 µm were inoculated with varying proportions of producer and cross-feeder cells but constant inoculation density (1∶1). In the x-axis, 0 means that the two microcolonies were inoculated with equal number of cells of species A and B and represents s∼0, whereas 1 means clonal microcolonies at inoculation, and therefore s = 1. An increment of 0.1 means a 5% increase (or decrease) in the number of cells of species A (or species B) inoculated in each microcolony. Data represent producer segregation index at inoculation (white circles), and after 12 and 96 hours growth (grey and black dots, respectively). Data are the mean of 3 replicates and error bars are the SD of the mean. A, D, low by-product toxicity; B, E, intermediate by-product toxicity; C, F, high by-product toxicity. (TIFF) [file pcbi.1003398.s006.tiff]

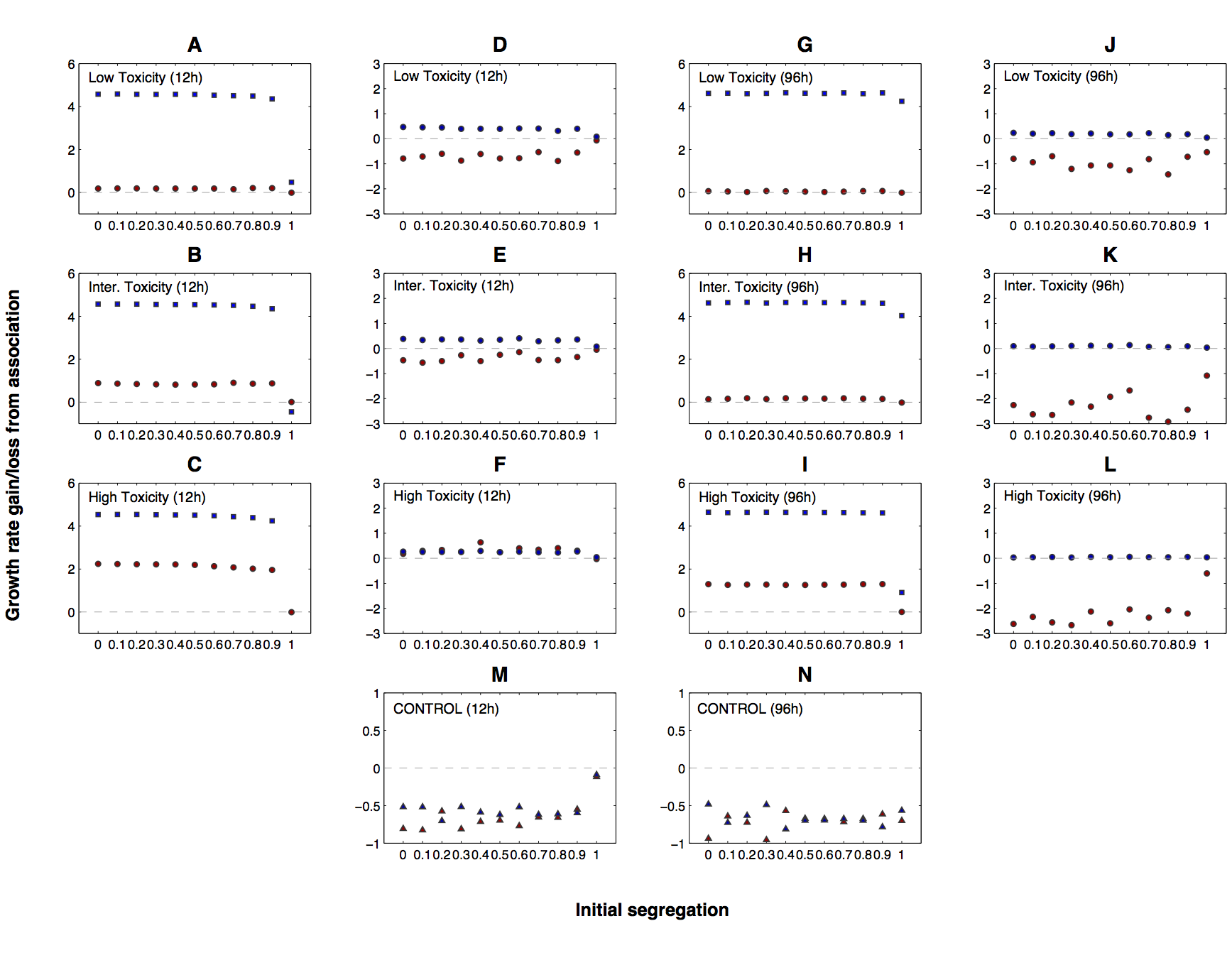

Supplement: Figure S7 — Costs and benefits of association for varying degree of intermixing at inoculation. A–C, G–I. Obligate cross-feeding, after 12 h and 96 h growth, respectively. D–F, J–L. Facultative cross-feeding (A−BfacI) after 12 h and 96 h growth, respectively. M–N. Control, after 12 h and 96 h growth, respectively. Measured as log(X co/X mono) where X co and X mono represent growth rate in coculture and monoculture, respectively (for growth rate calculation see Methods). To note that obligate cross-feeder growth rate is measured as log(Bco) because the obligate cross-feeder cannot grow in monoculture. Positive and negative values indicate a net gain and loss from association, respectively. Red dots represent producer, blue squares represent obligate cross-feeder, and blue dots represent facultative cross-feeder. In the control scenario, the two types are identical except for their color, i.e. red-tagged cells or blue-tagged cells. See legend fig. 3 for details on inoculation conditions. (TIFF) [file pcbi.1003398.s007.tiff]

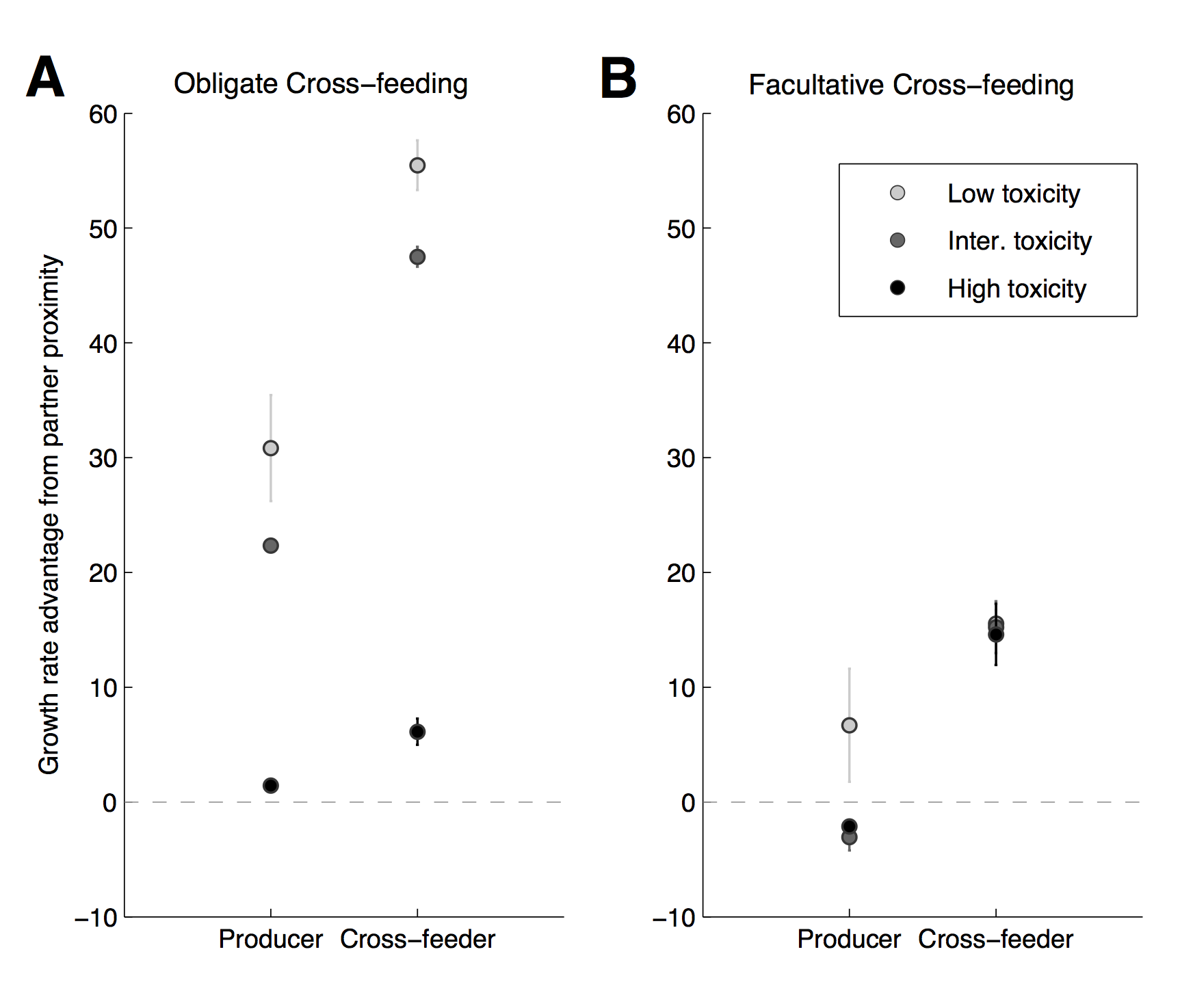

Supplement: Figure S8 — Effect of interspecific partner proximity at seeding. A. Obligate cross-feeding. B, Facultative cross-feeding (A−BfacI). Growth rate advantage is measured as the difference between the growth rate of a producer (cross-feeder) growing close to a cross-feeder (producer) and the growth rate of a producer (cross-feeder) growing far from a cross-feeder (producer). Thus, positive values mean a growth rate advantage from interspecific partner proximity whereas negative values mean a growth rate disadvantage from interspecific partner proximity. Boundaries on the sides of the domain are permeable to the by-product and non cyclic. Data represent 120 hours growth, are the mean of 3 replicates, and error bars are the SD of the mean. (TIFF) [file pcbi.1003398.s008.tiff]

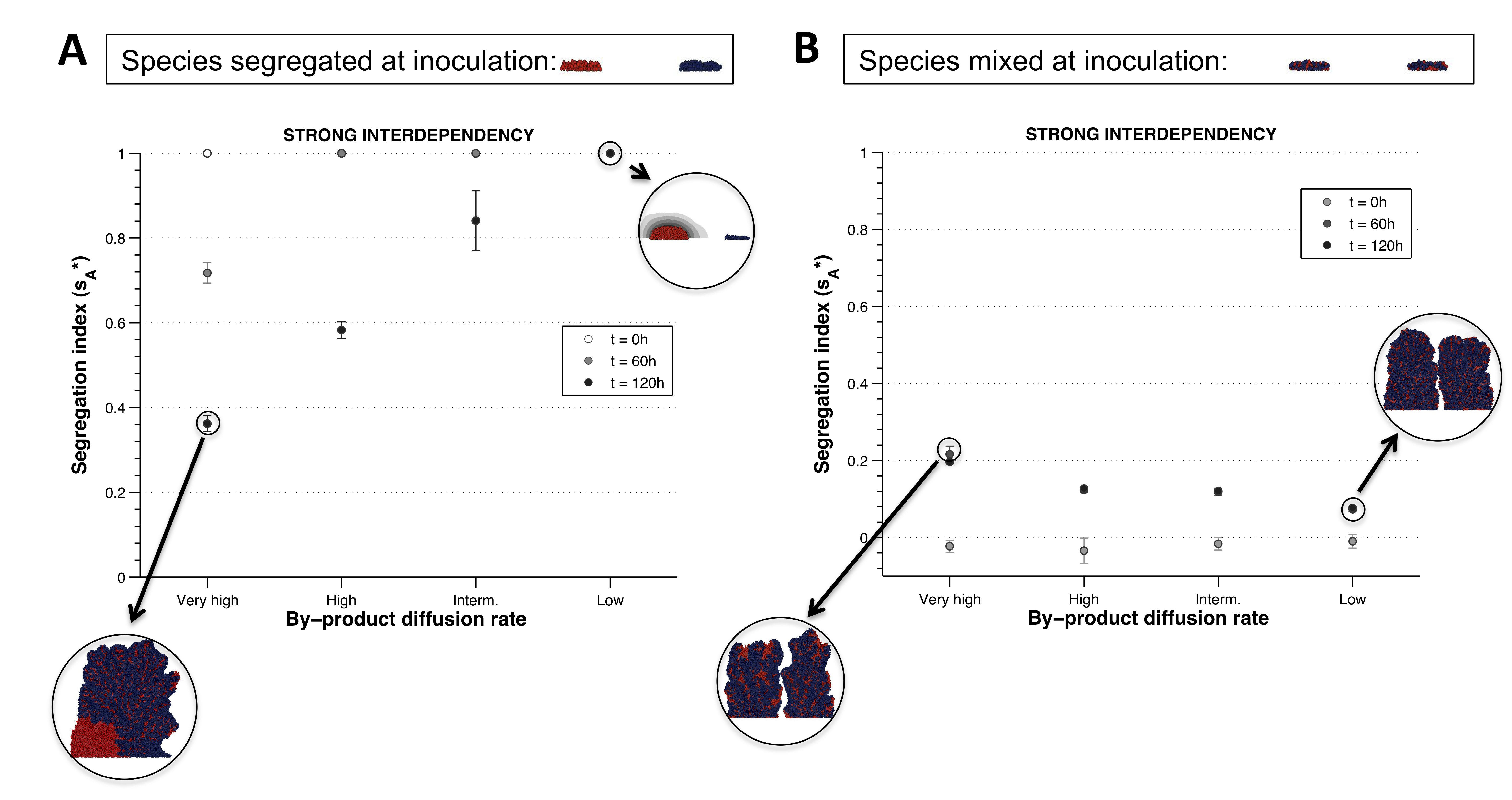

Supplement: Figure S10 — Effect of by-product diffusion rate on strongly interdependent communities given initial segregation A, and, initial mixing B. Producer segregation index (sA*) was measured for a neighbourhood of 5 um (see legend fig. 3 and Methods section for further details). Given the strong mixing pattern of strongly interdependent communities, here we decreased the size of the neighbourhood to measure spatial structuring even more locally. By-product diffusion rates are [10DE; 1.4DE; DE; 0.14DE] from very high to low, respectively (see Table S2). Data are the mean of 3 replicates. (TIFF) [file pcbi.1003398.s010.tiff]

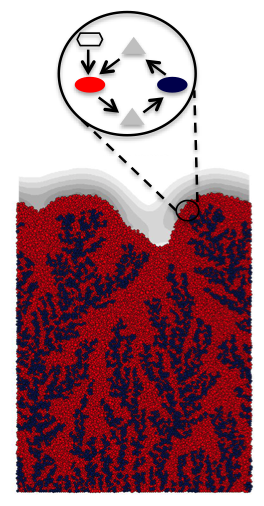

Supplement: Figure S11 — Emergent branching pattern of a two-species community involved in bidirectional nutritional benefits (cross-feeding). The schematic illustrates the metabolic interaction scenario. Specifically, the two species are identical in their cross-feeding capabilities but species A (red) is also able to use the limiting nutrient (hexagon) while species B (blue) is obligate on species A's by-product for growth. By-products are represented by triangles. Biofilm image after 60 h growth. (TIFF) [file pcbi.1003398.s011.tiff]
