# Supplementary material for: Metabolic and Demographic Feedbacks Shape the Emergent Spatial Structure and Function of Microbial Communities
Source: PLoS Comput Biol. 2013 Dec 26;9(12):e1003398. doi: 10.1371/journal.pcbi.1003398 (PMC3873226; doi:10.1371/journal.pcbi.1003398)
Supplement: Table S1 — Reactions and respective stoichiometry of biological processes used in the models. (PDF) [file pcbi.1003398.s012.pdf]

Table S1. Reactions and respective stoichiometry of biological processes used in the models

Process	Solute		Biomass		Rate expression
	R	***E***	$\boldsymbol{X_A}$	$\boldsymbol{X_B}$	
Growth of producer, A	-1	$Y_{E,A}$	$Y_{R,A}$	-	$\mu_{\max,A}\dfrac{R}{K_{R,A}+R}\dfrac{K_{i,E}}{K_{i,E}+E}X_A$
Growth of cross-feeder, B	-1	-1	-	$Y_{R,B}+Y_{E,B}$	$\mu_{\max,B}\left(\dfrac{R}{K_{R,B}+R}+\dfrac{E}{K_E+E}\right)X_B$
