# Supplementary material for: Metabolic and Demographic Feedbacks Shape the Emergent Spatial Structure and Function of Microbial Communities
Source: PLoS Comput Biol. 2013 Dec 26;9(12):e1003398. doi: 10.1371/journal.pcbi.1003398 (PMC3873226; doi:10.1371/journal.pcbi.1003398)
Supplement: Table S2 — Model parameters. (PDF) [file pcbi.1003398.s013.pdf]

Table S2. Model parameters

Symbol	Description	Value	Unit
$\mu_{max, A}$	Maximum cell growth rate of species A	1^*	h^{-1}
$\mu_{max, B}$	Maximum cell growth rate of species B	1^*	h^{-1}
$Y_{R, A}$	Biomass yield of species A on R	0.5^*	$g \cdot g^{-1}$
$Y_{E, A}$	Yield of E produced per R consumed	2	$g \cdot g^{-1}$
$Y_{R, B}$	Biomass yield of species B on R	0.125 (B_{facW})<br>0.25 (B_{facI})<br>0.375 (B_{facS})<br>0.5 (B_{ncf})	$g \cdot g^{-1}$
$Y_{E, B}$	Biomass yield of species B on E	0.125 (B_{facS})<br>0.25 (B_{facI})<br>0.375 (B_{facW})<br>0.5 (B_{obl})	$g \cdot g^{-1}$
$K_{R, A}$	Species A half-saturation constant for R	$3.5 \times 10^{-5\,*}$	$g \cdot L^{-1}$
$K_{R, B}$	Species B half-saturation constant for R	3.5×10^{-4}	$g \cdot L^{-1}$
K_E	Species B half-saturation constant for E	3.5×10^{-5} (B_{fac})<br>3.5×10^{-6} (B_{obl})	$g \cdot L^{-1}$
$K_{i, E}$	Half-saturation inhibition constant of E on species A	3.5×10^{-2} (low tox.)<br>3.5×10^{-3} (inter tox.)<br>3.5×10^{-4} (high tox.)	$g \cdot L^{-1}$
R_{Bulk}	Concentration of R in the bulk	0.125^*	$g \cdot L^{-1}$
D_R	R diffusivity	$9.6 \times 10^{-7\,*}$	$m^2 \cdot day^{-1}$
D_E	E diffusivity	$7.2 \times 10^{-6\,*}$	$m^2 \cdot day^{-1}$

*Values from [1]

References

1. Mitri S, Xavier JB, Foster KR (2011) Social evolution in multispecies biofilms. Proc Natl Acad Sci U S A 108 Suppl 2: 10839-10846.
